# Posterior semicircular canal ossification following acute vestibular loss mimicking inferior vestibular neuritis: A case report

**DOI:** 10.3389/fneur.2022.1015555

**Published:** 2022-10-17

**Authors:** Francesco Comacchio, Andrea Castellucci

**Affiliations:** ^1^ENT Unit, Regional Vertigo Specialized Center, University Hospital of Padova, Sant'Antonio Hospital, Padova, Italy; ^2^ENT Unit, Department of Surgery, Azienda USL–IRCCS di Reggio Emilia, Reggio Emilia, Italy

**Keywords:** acute vestibular loss, labyrinthine ossification, video head impulse test, labyrinthine ischemia, case report, inferior vestibular neuritits

## Abstract

Vestibular neuritis (VN) mostly involves the superior vestibular nerve. Isolated inferior vestibular neuritis (IVN) has been more rarely described. The diagnosis of IVN is based on an abnormal head impulse test (HIT) for the posterior semicircular canal (PSC), pathological cervical vestibular-evoked myogenic potentials (C-VEMPs), and spontaneous downbeat nystagmus consistent with acute functional loss of inner ear sensors lying within the inferior part of the labyrinth. HIT for both lateral and superior semicircular canals is normal, as are ocular VEMPs and bithermal caloric irrigations. The etiology of IVN is debated since peripheral acute vestibular loss with a similar lesion pattern can often be associated with ipsilesional sudden hearing loss (HL). Viral inflammation of vestibular nerves is considered the most likely cause, although reports suggest that VN usually spares the inferior division. On the other hand, an ischemic lesion involving the terminal branches of the common cochlear artery has been hypothesized in cases with concurrent HL. Debated is also the lesion site in the case of IVN without HL since different instrumental patterns have been documented. Either isolated posterior ampullary nerve involvement presenting with selective PSC functional loss on video-HIT, or only saccular lesion with isolated ipsilesional C-VEMPs impairment, or inferior vestibular nerve damage (including both saccular and posterior ampullary afferents) exhibiting an impairment of both C-VEMPs and PSC-HIT. We report an interesting case of a patient with an acute vestibular loss consistent with IVN without HL who developed a PSC ossification on follow-up, questioning the viral origin of the lesion and rather orienting toward an occlusion of the posterior vestibular artery. To the best of our knowledge, this is the first report of PSC ossification after a clinical picture consistent with IVN.

## Introduction

Vestibular neuritis (VN) represents the most common cause of peripheral acute vestibular loss (AVL) (1) and usually involves either the superior vestibular nerve or both superior and inferior divisions (2–4). In rare cases, an isolated inferior vestibular neuritis (IVN) may occur (3, 5–8), while bilateral involvement has been only anecdotally reported (9, 10). The diagnosis of IVN is based on an abnormal head impulse test (HIT) for the posterior semicircular canal (PSC), pathological cervical-vestibular evoked myogenic potentials (C-VEMPs), and spontaneous downbeat nystagmus (DBN) with variable torsional components aligning with the plane of the affected PSC, consistent with an acute functional loss of labyrinthine sensors lying within the inferior part of the inner ear (5, 8). Since the receptors innervated by the superior vestibular branch are spared, HIT for the horizontal (HSC) and superior semicircular canals (SSC) is normal, as well as ocular-VEMPs (O-VEMPs) and bithermal caloric stimulation. The etiology of VN is still debated. A viral inflammation involving either the whole vestibular nerve or one of its divisions is the most likely cause (1, 2, 4, 11), even though selective ischemia of the terminal branches of the internal auditory artery has been demonstrated to result in similar clinical pictures and instrumental patterns (12–14). However, it has also been reported that VN often spares the inferior vestibular branch and that AVL exhibiting a clinical picture consistent with IVN can be non-rarely associated with sudden hearing loss (HL), indicating a vascular origin of the lesion rather than neural damage (15–18). Moreover, various degrees of involvement of the end-organs innervated by the inferior vestibular nerve (i.e., PSC and saccule) have been described in the case of IVN without HL. It has been reported that either an isolated acute PSC failure with pathological HIT for the affected PSC and normal C-VEMPs, or a selective saccular lesion presenting with impaired C-VEMPs and normal vestibulo-ocular reflex (VOR) gain for the PSC, or eventually a sudden hypofunction of both saccular and posterior ampullary afferents presenting with simultaneous C-VEMPs and PSC VOR-gain impairment (5, 8, 17–20). Only the latter combination properly refers to IVN, as the other aforementioned lesion patterns could be attributed to other selective intralabyrinthine lesions. Conversely, in the case where AVL involving both saccular and PSC afferents is associated with sudden HL, a vascular occlusion of the common cochlear artery (CCA) or one of its branches should always be considered as the most likely underlying pathomechanism (14–18), as the lack of cochlear symptoms should be needed to fulfill VN diagnostic criteria (4). We herein report an interesting case of AVL without HL consistent with IVN, presenting with spontaneous DBN, PSC hypoactivity on video-HIT, and absent C-VEMPs on the affected side, that developed ipsilesional PSC ossification after a brief follow-up questioning the viral origin of the lesion and rather orienting toward an occlusion of the posterior vestibular artery (PVA). To the best of our knowledge, this is the first case of labyrinthine ossification following an AVL mimicking IVN.

## Case description

**Figure 1:**
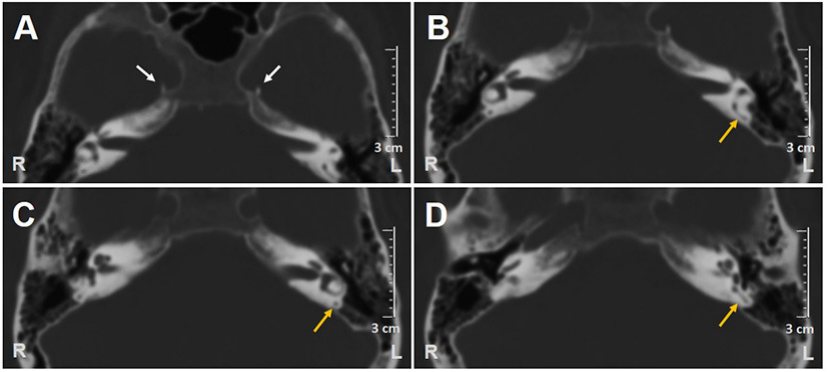
Axial scans of brain CT show **(A)** bilateral carotid siphon calcifications (white arrows) and **(B–D)** patent left PSC (yellow arrows). CT, computed tomography; L, left; PSC, posterior semicircular canal; R, right.

**Figure 2:**
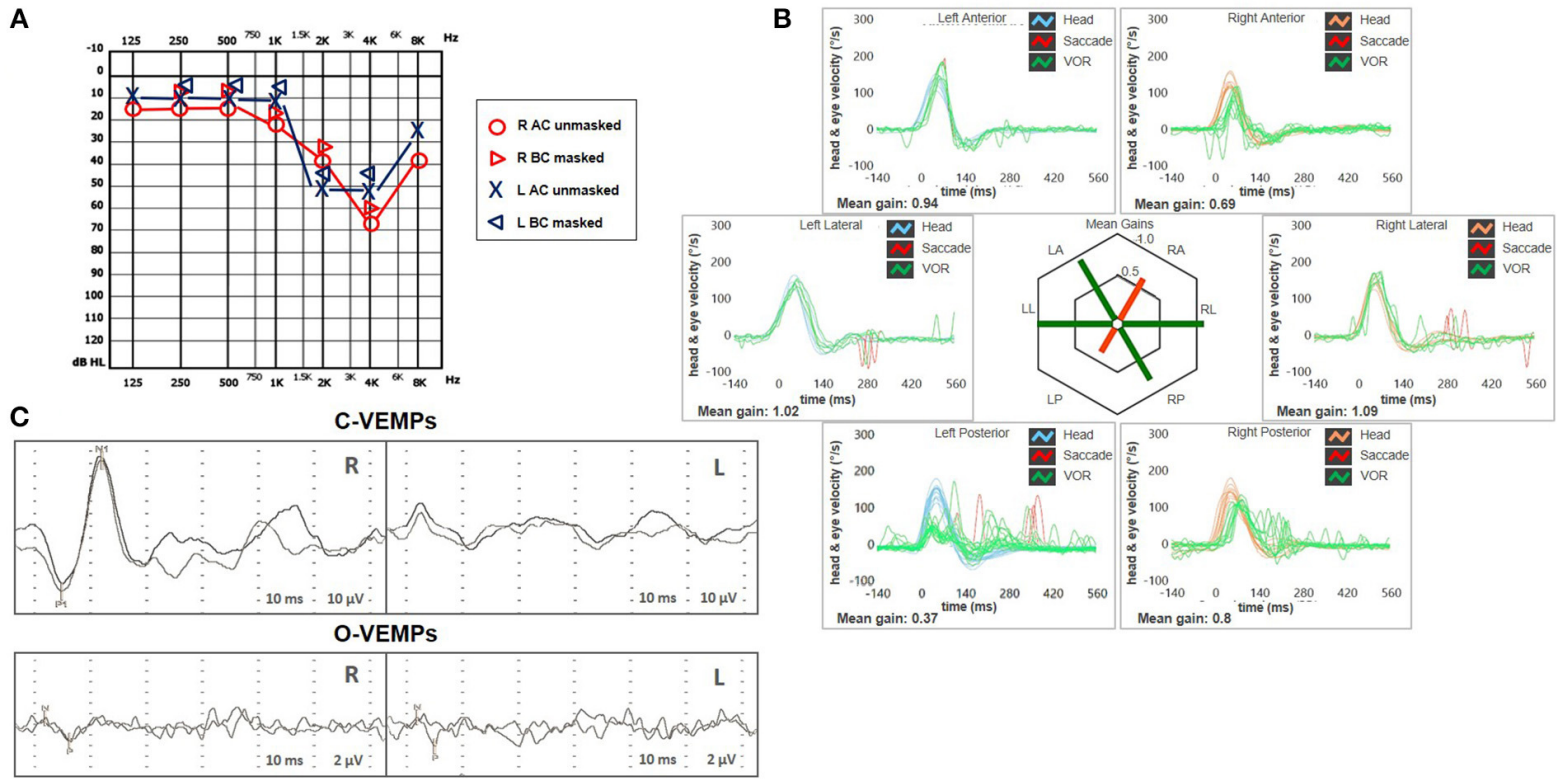
Presenting scenario including **(A)** Pure-tone audiometry exhibiting bilateral high-frequency sensorineural hearing impairment, slightly greater on the right side. **(B)** Video-HIT was performed using a portable high-frame-rate video-oculography device (ICS Impulse, Natus Medical Inc, Denmark). Blue lines represent head impulses exciting left canals, orange lines correspond to impulses for right canals, green lines represent eye movements induced by the activation of VOR following each impulse, and red lines correspond to corrective saccades. The mean value of VOR-gain (eye velocity/head velocity) is reported for each canal. The hexagonal plot in the center of the figure summarizes the mean VOR gains for each canal; normal gains are shown in green, and deficient gains are in red. Gains are considered normal if> 0.8 for lateral canals and> 0.7 for vertical canals. A severe VOR-gain impairment for the left PSC (0.37) with mainly overt saccades can be observed, and a slight reduction of contralateral ASC VOR-gain (0.69). **(C)** C-VEMPs (above) and O-VEMPs (below) for air-conducted sounds recorded with a 2-channel evoked potential acquisition system (Viking, Nicolet EDX, CareFusion, Germany). Potentials were measured by delivering tone bursts (intensity: 100 dB nHL, frequency: 500 Hz, duration: 8 ms, stimulation rate 5 Hz) *via* headphones. The recording system used an EMG-based biofeedback monitoring method to minimize variations in muscle contractions and VEMPs amplitudes. Each stimulus was retested to assess the reproducibility of responses. For C-VEMPs, right and left lines correspond to myogenic responses (p1–n1) recorded on the right and left SCM muscle (i.e., right and left saccular responses), respectively. For O-VEMPs, being crossed responses, right and left lines representing potentials (n–p) are recorded under the left and right eye (i.e., right and left utricular responses), respectively. VEMP testing revealed normal responses on the right side (105.5 μV) and absent potentials on the left, whereas symmetrical amplitudes for ocular VEMPs (R: 4.4 μV and L: 5.7 μV) could be detected. AC, air-conduction; ASC, anterior semicircular canal; BC, bone-conduction; C, cervical; HIT, head impulse test; L, left; LA, left anterior; LL, left lateral; LP, left posterior; O, ocular; PSC, posterior semicircular canal; R, right; RA, right anterior; RL, right lateral; RP, right posterior; SCM, sternocleidomastoid; VEMPs, vestibular evoked myogenic potentials; VOR, vestibulo-ocular reflex.

**Figure 3:**
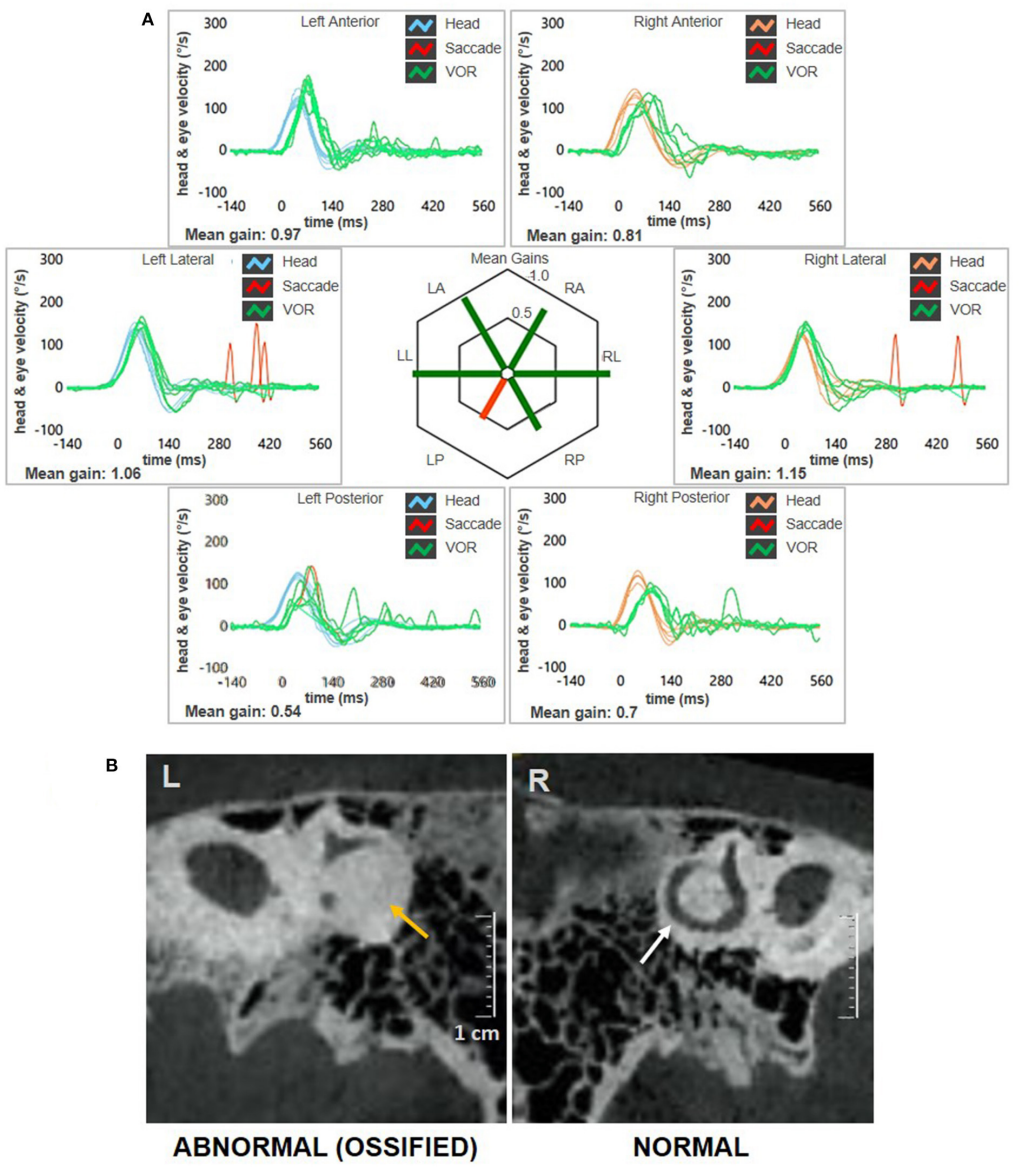
Instrumental picture observed at 3-months follow-up. **(A)** Video-HIT exhibited persistent selective loss for the left PSC VOR-gain (0.54) with only covert saccades. The affected canal VOR-gain is slightly increased compared to presenting values. **(B)** Cone-bream CT scans of the temporal bones with parasagittal reconstructed images along the Stenver plane detecting normally patent right-sided PSC (white arrow) and a near-complete PSC ossification on the left side (yellow arrows). CT, computed tomography; HIT, head impulse test; L, left; LA, left anterior; LL, left lateral; LP, left posterior; PSC, posterior semicircular canal; R, right; RA, right anterior; RL, right lateral; RP, right posterior; VOR, vestibulo-ocular reflex.

A 62-year-old male with acute vertigo, unsteadiness, nausea, and vomiting was admitted to the emergency department. He denied recurrent headaches and significant auditory symptoms. His clinical history was consistent with hypercholesterolemia and acid reflux. Six years earlier, he had presented with a sudden flat right-sided sensorineural HL that recovered fully on oral steroids. On admission, neurological evaluation and bedside oculomotor testing (including saccades, smooth pursuit, and test of skew) excluded signs of central nervous system (CNS) involvement. Both blood pressure and pulse rate were within normality ranges, and electrocardiography was unremarkable. Only slight signs of bilateral carotid siphon calcifications were noted on a standard brain CT scan (Figure 1A), whereas serology for SARS-CoV-2 was negative. Otoneurological examination with monocular video-Frenzel goggles detected spontaneous vertical DBN inhibited by visual fixation while slightly enhanced by 100 Hz-mastoid vibrations and head shaking. In addition, horizontal right beating components could be detected in a supine position, whereas neither bilateral gaze nor hyperventilation tests changed spontaneous oculomotor patterns (Supplementary Video 1). No corrective saccades could be detected on the horizontal bedside HIT, and moderate-to-severe ataxia was found in the Romberg test. Therefore, the patient was scheduled for an additional CT scan 48 hours later to rule out a posterior fossa stroke. New imaging still excluded abnormal findings in the brainstem and the cerebellum. Despite low-resolution scans and thick slices, normal patency and morphology of the inner ear structures could be verified in temporal bone scans (Figures 1B–D). The patient was then submitted to an extensive instrumental assessment. On micro-otoscopy, tympanic membranes were unremarkable, impedance audiometry was normal, while pure tone audiometry detected bilateral sensorineural high-frequency HL, with noise-induced components slightly greater in the right ear, comparable to the previous audiogram (Figure 2A). Video-HIT documented a severe impairment for the left PSC VOR-gain (0.37) with mainly overt saccades (Figure 2B), whereas bithermal caloric irrigations were within normality ranges. While O-VEMPs were symmetrical, C-VEMPs revealed no responses on the left side, consistent with saccular impairment (Figure 2C). A gadolinium-enhanced brain MRI performed the following week only showed signs of periventricular leukoaraiosis, with normal findings in the posterior fossa. Therefore, left-sided IVN was diagnosed according to clinical and instrumental findings. Despite a first therapeutic approach including steroids, vestibular suppressants, and antiemetic drugs followed by a 3-month pharmacological treatment enhancing vestibular compensation (Betahistine 24 mg twice a day and Citicoline 1 g a day), the patient kept complaining of continuous unsteadiness and oscillopsia, particularly walking down the stairs. He was then sent to a tertiary referral center for vestibular dysfunction. The persistence of slight spontaneous DBN inhibited by visual fixation was ascertained on video-Frenzel examination. Skull vibration greatly enhanced spontaneous nystagmus and a new video-HIT confirmed the left PSC hypoactivity, despite a slight VOR-gain improvement (0.54) (Figure 3A). A cone-beam CT scan of the temporal bones was scheduled to exclude possible semicircular canal dehiscences. Surprisingly, a sub-total left PSC ossification was found (Figure 3B), consistent with ischemic damage in the labyrinthine territory supplied by the PVA. Prophylactic treatment with acetylsalicylic acid was then suggested, and the patient was advised to undergo vestibular rehabilitation therapy. Written informed consent was obtained from the patient to publish this case report, including all data and images.

## Discussion

**Figure 4:**
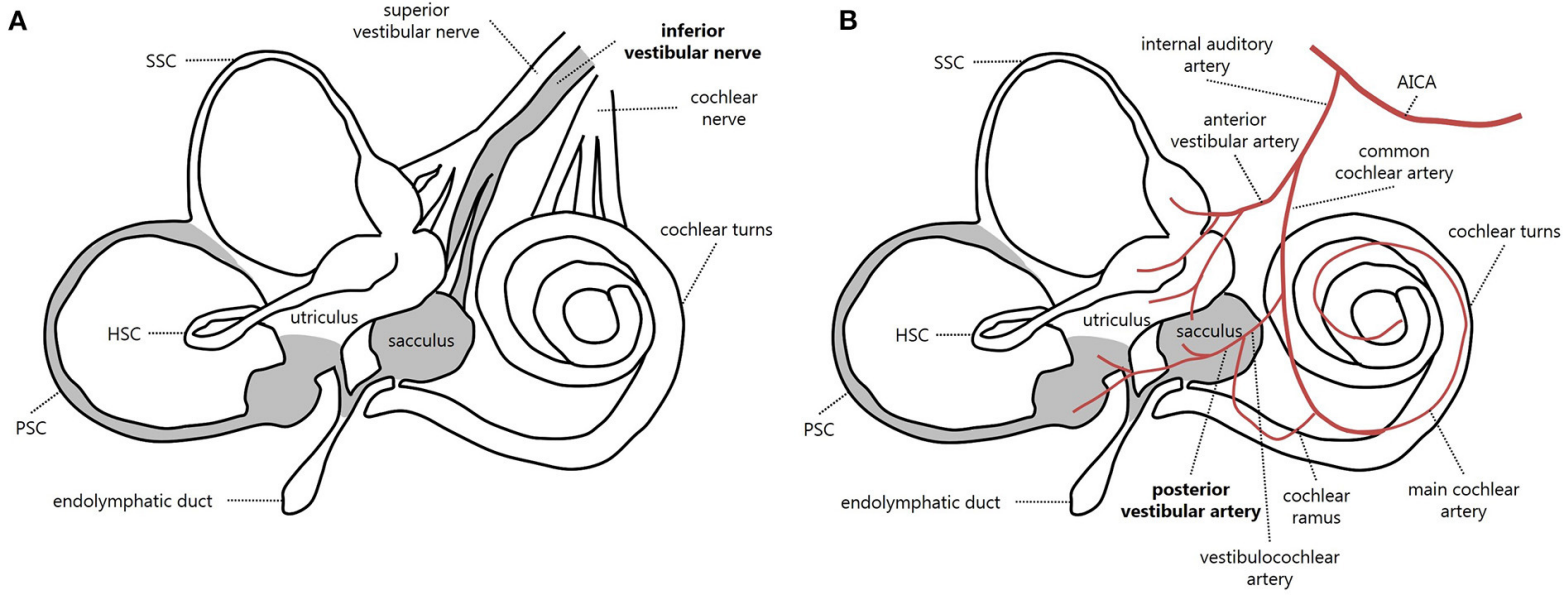
Schematic representation of inner ear innervation **(A)** and vascular supply **(B)** showing possible pathomechanisms accounting for an acute loss of PSC and saccular function [modified from (21)]. Labyrinthine receptors innervated by the inferior vestibular nerve and mainly supplied by the posterior vestibular artery are gray. AICA, anterior-inferior cerebellar artery; HSC, horizontal semicircular canal; PSC, posterior semicircular canal; SSC, superior semicircular canal.

The vestibular nerve is composed of two branches, i.e., the superior and the inferior vestibular nerve. While the former is composed of the lateral and anterior ampullary nerves and the utricular nerve, the inferior vestibular nerve is composed of the singular nerve (or posterior ampullary nerve) and the saccular nerve (21) (Figure 4A). IVN is considered a rare entity, with a possible prevalence of 1.3–18% among overall VN cases, depending on different inclusion criteria and available instrumental battery (3, 7, 8, 22–25). Diagnosis is often challenging due to spontaneous vertical nystagmus and lack of corrective saccades on horizontal HIT, which is supposed to guide toward an acute CNS disorder (5, 26). Before the widespread availability of modern tools for vestibular testing enabling a rapid assessment of both otolith and ampullary receptors, the correct diagnosis of IVN could only rely on the identification of the plane aligning with spontaneous DBN and reduced VOR-gain measures for the PSC using a scleral search coil, in association with normal findings on caloric irrigations (3, 5). Only in recent years has the combined use of the video-HIT and VEMPs enabled clinicians to measure the high-frequency response of all labyrinthine receptors, even in an acute setting, allowing reliable detection of isolated dysfunctions and lesion patterns peculiar to specific pathomechanisms (8, 16–18, 24, 25).

The clinical presentation of IVN, fairly atypical for AVL and rather addressing a CNS lesion, at first sight, might account per se for a possible underestimation of the actual incidence of the disorder (3, 5, 22, 26). Moreover, the rare involvement of the inferior vestibular nerve in VN has been mostly explained by anatomic differences rendering the superior division more vulnerable to entrapment during inflammatory swelling and ischemia than the singular and inferior vestibular nerves. They include the greater length of the superior vestibular division, the narrower canal lumen, and the larger percentage of bony spicules occupying the channel where the superior nerve and its vascular supply run (27, 28). Moreover, in human temporal bone specimens, anastomoses between the facial nerve (representing an additional pathway for viral spread) and the superior vestibular nerve have been more commonly found than the inferior branch (29).

In the reported case, the presenting clinical scenario perfectly overlapped an AVL involving the inferior vestibular nerve. In fact, spontaneous DBN associated with a selective loss of left PSC and ipsilaterally absent cVEMPs with no auditory impairment could likely be due to a left IVN. Moreover, both the enhancement of DBN after head shaking and skull vibration and the paretic horizontal components elicited in the supine position have also been described in other reports of IVN (7, 8, 18, 22).

Despite the association of spontaneous DBN with a selective loss of PSC on vHIT has been reported in other conditions, such as a canalith jam involving the posterior canal (30, 31) or Meniere's disease in the ictal stage (32, 33), neither a sign of benign paroxysmal positional vertigo nor fluctuating HL have been documented before and after our evaluation; therefore, other inner ear disorders than IVN were excluded. Nevertheless, hyperventilation did not affect spontaneous nystagmus in our case. This finding seemed not to be perfectly in line with the typical directional and amplitude changes of spontaneous nystagmus described in the acute stage of VN, where ionic alterations due to a reduction of paCO_2_ should result in a transient improvement of the neuronal excitability of the affected side (34).

The great debate is, therefore, focused on the etiology, particularly the pathomechanisms underlying IVN. Generally, it seems well accepted that VN is caused by a viral infection or viral reactivation, although a previous upper respiratory tract infection is mostly lacking in the patient's history. Furthermore, the pattern of clinical recovery from IVN seems somehow different from superior or total VN, exhibiting some controversial issues. In fact, it has been documented how the time course of IVN is shorter than other VN, with a faster recovery for afferents running in the inferior branch (22), while, in contrast, the PSC seems to recover more slowly than other semicircular canals in VN according to other descriptions (23). On the other hand, it has been well documented how clinical and instrumental patterns consistent with AVL could be due to selective ischemia of the terminal branches of the internal auditory artery (12–14). In particular, presenting scenarios overlapping IVN associated with cochlear symptoms has oriented the aetiologic hypothesis toward a possible occlusion of the CCA (15–18).

Our finding of an early ossification of the PSC after a clinical picture consistent with IVN without HL increases the aetiologic dilemma. Herpes Simplex Virus (HSV) represents one of the most commonly associated viral agents accounting for VN (11, 35). Nevertheless, histopathologic examination of the temporal bones in patients with previous VN revealed neural atrophy and variable degeneration of the labyrinthine neuroepithelium but no signs of ossification (1, 21, 36). Similarly, in experimental HSV labyrinthitis, Nomura et al. (37) described an involvement of both the cochlea and the posterior labyrinth when the virus was inoculated *via* the middle ear, while ossification of the semicircular canals was not described. Moreover, Himmelein et al. (29), studying the presence of HSV in the vestibular nerves and ganglion, rarely found the virus itself in the IVN, and a complete absence of latent mRNA of HSV in the IVN was ascertained.

Labyrinthitis ossificans (LO) is a pathologic process resulting from the progressive new bone formation (i.e., ossification) within the membranous labyrinth, leading to sensorineural HL in the majority of cases. Suppurative infection of the middle/inner ear, otosclerosis, head trauma, surgery of the temporal bone, malignant infiltration of the inner ear, autoimmune processes, meningitis, sickle cell disease, and other genetic disorders such as Fabry's disease represent the most common causes (21). Selective ossification of a single semicircular canal is very rare. Castellucci et al. (38) were recently able to demonstrate a filling defect in the PSC on MRI, consistent with early fibrosis of the canal, in a patient with a sudden cochleovestibular loss likely due to CCA ischemia. A labyrinthine ossification was clearly demonstrated two months after vascular inner ear occlusion in guinea pigs by Kimura and Perlman (39, 40). Therefore, ischemia also represents a possible etiologic factor resulting in LO.

The inner ear is supplied by the internal auditory artery, which branches from the anterior-inferior cerebellar artery and divides into two main terminal branches: the anterior vestibular artery and the CCA. Whereas the first mostly supplies the utricle and both HSC and SSC, the latter mainly serves the cochlea, saccule, and PSC. In turn, the CCA divides into the vestibulocochlear artery, which serves the PSC, saccule, and cochlear basal turn. The main cochlear artery supplies the rest of the cochlear neuroepithelium. Finally, the PVA is generated from the vestibulocochlear artery and provides blood supply to both the PSC and saccule (21, 39–41) (Figure 4B). Therefore, the clinical and instrumental effects of PVA occlusion perfectly overlap with IVN. While in the case described by Castellucci et al. (38), an occlusion of CCA was suggested to explain the involvement of the lower labyrinthine structures and PSC fibrosis, a possible PVA terminal occlusion seems to represent the possible site of vascular lesion in our case, resulting in acute PSC and saccular impairment without auditory symptoms. Since our patient denied previous infections, trauma, surgery, and middle/inner ear dysfunctions other than previous contralateral sudden sensorineural HL, most conditions accounting for LO could be reasonably excluded, while inner ear ischemia seems to represent the most likely pathomechanism. Kimura and Pearlman (40) found that the PSC ampulla was more often involved in ischemia than HSC and SSC ampullae because the particular vessel coagulated. Fibrosis was noted after two weeks and ossification after two months, and in our case, the ossification was similarly detected after a few months.

To the best of our knowledge, this is the first report showing LO involving an inner ear structure based on CT findings before and after an AVL mimicking IVN. Since it is well known how vascular lesions of the inner ear may precede a major stroke involving CNS (42, 43), otoneurologists should be aware of the possibility of labyrinthine ischemia and treat patients accordingly. Therefore, we encourage clinicians to routinely exclude ischemic damage to the inner ear with proper investigations, including imaging, even when clinical presentation and instrumental pattern indicate a VN, particularly when symptoms do not improve over time as expected.

Besides detecting PSC ossification, cone-beam CT scans also allowed us to rule out canal dehiscence. In fact, it has been well documented how vertical canal dehiscence might account for vertical/torsional nystagmus after skull vibrations and reduced VOR-gain value for the affected canal on video-HIT (44–46). Nevertheless, in the case of dehiscence, both C-VEMPs and O-VEMPs should have exhibited enhanced potentials and reduced thresholds on the affected side, besides ipsilesional low-frequency conductive HL, unlike the case herein described.

Another interesting finding in our case is that the VOR-gain value for the affected left-sided PSC was not zero on video-HIT but rather improved from 0.37 to 0.54 in a few months despite a complete ossification of the canal. Similarly, VOR-gain values for the contralesional SSC were slightly impaired at presentation despite the right ear not being affected. These findings confirm that canal VOR-gain values, as measured by the video-HIT gain, reflect the loss of function of the ampullary receptor and are related to the function of the paired semicircular canal aligning with the stimulation plane (47). The reason is that the excitatory and inhibitory responses from the right and left paired canals work in a push-pull manner to generate compensatory eye movements, and video-HIT measurements are probably influenced by additional compensatory mechanisms that still need to be fully understood. The lack of frequent saccades for the affected PSC-HIT likely reflects poor visual compensation enhancing the patient's unsteadiness. On the other hand, low-amplitude refixation saccades detected after the head impulses for the contralesional PSC could be either related to a previous subclinical vestibular involvement, likely concomitant with the right-sided sudden sensorineural HL, or ascribed to an age-related physiological impairment (48).

## Conclusions

In conclusion, the case herein described represents the first observation of PSC ossification after a clinical picture consistent with IVN. Owing to an extensive assessment, including instrumental tests measuring labyrinthine function and imaging, it could be possible to identify the labyrinth as the site of the lesion, highlighting the possible role of PVA occlusion. In the case of AVL without HL consistent with IVN, a vascular pathomechanism should always be considered in the differential diagnosis.

## Data availability statement

The original contributions presented in the study are included in the article/Supplementary material, further inquiries can be directed to the corresponding authors.

## Ethics statement

Written informed consent was obtained from the patient to publish this case report, including all data and images.

## Author contributions

FC: conceptualization, investigation, data acquisition and interpretation, and original draft preparation. AC: investigation, data acquisition, interpretation, images and artwork, and manuscript review. All authors approved the final version of the manuscript.

## Conflict of interest

The authors declare that the research was conducted in the absence of any commercial or financial relationships that could be construed as a potential conflict of interest.

## Publisher's note

All claims expressed in this article are solely those of the authors and do not necessarily represent those of their affiliated organizations, or those of the publisher, the editors and the reviewers. Any product that may be evaluated in this article, or claim that may be made by its manufacturer, is not guaranteed or endorsed by the publisher.

## Abbreviations

AVL: acute vestibular loss
CCA: common cochlear artery
CNS: the central nervous system
CT: computed tomography
DBN: downbeat nystagmus
HIT: head impulse test
HL: hearing loss
HSC: horizontal semicircular canal
HSV: Herpes Simplex Virus
IVN: inferior vestibular neuritis
LO: labyrinthitis ossificans
MRI: magnetic resonance imaging
PSC: posterior semicircular canal
PVA: posterior vestibular artery
SSC: superior semicircular canal
VEMPs: vestibular-evoked myogenic potentials
VN: vestibular neuritis
VOR: vestibulo-ocular reflex.
